# A New HPLC-MS Method for Measuring Maslinic Acid and Oleanolic Acid in HT29 and HepG2 Human Cancer Cells

**DOI:** 10.3390/ijms160921681

**Published:** 2015-09-09

**Authors:** Juan Peragón, Eva E. Rufino-Palomares, Irene Muñoz-Espada, Fernando J. Reyes-Zurita, José A. Lupiáñez

**Affiliations:** Biochemistry and Molecular Biology Section, Department of Experimental Biology, University of Jaén, Campus Las Lagunillas, E-23071 Jaén, Spain; E-Mail: mebeverina@gmail.com; Department of Biochemistry and Molecular Biology I, Faculty of Sciences, University of Granada, Campus Fuentenueva, E-18001 Granada, Spain; E-Mails: evaevae@ugr.es (E.E.R.-P.); ferjes@ugr.es (F.J.R.-Z.); jlcara@ugr.es (J.A.L.)

**Keywords:** maslinic acid, oleanolic acid, HPLC-MS, HT29, HepG2

## Abstract

Maslinic acid (MA) and oleanolic acid (OA), the main triterpenic acids present in olive, have important properties for health and disease prevention. MA selectively inhibits cell proliferation of the HT29 human colon-cancer cell line by inducing selective apoptosis. For measuring the MA and OA concentration inside the cell and in the culture medium, a new HPLC-MS procedure has been developed. With this method, a determination of the amount of MA and OA incorporated into HT29 and HepG2 human cancer-cell lines incubated with different concentrations of MA corresponding to 50% growth inhibitory concentration (IC_50_), IC_50/2_, IC_50/4_, and IC_50/8_ has been made. The results demonstrate that this method is appropriate for determining the MA and OA concentration in different types of cultured cells and reveals the specific dynamics of entry of MA into HT29 and HepG2 cells.

## 1. Introduction

Maslinic acid [MA, (2α,3β)-2,3-dihydroxylolean-12-en-28-oic acid] and oleanolic acid (OA, 3β-hydroxylolean-12-en-28-oic acid) are two main pentacyclic triterpenic carboxylic acids found in the olive tree (*Olea europaea*) [1]. Pentacyclic triterpenes comprise a group of plant secondary metabolites that have important biological properties significant to human health. Recently, MA has been found to exert important effects such as growth stimulating [2,3,4,5,6] anti-oxidant [7,8,9,10,11], anti-inflammatory [12], anti-microbial and anti-viral [13,14,15], and even anti-tumoral [16,17,18,19,20]. With respect to the latter, it is of great interest that MA selectively inhibits cell proliferation in human colon-cancer HT29 cell line [17] by inducing apoptosis [16,17] and interfering with the normal cytoskeleton function [21]. The effects that the MA concentration in the cell culture exerts on growth survival has been well characterized and the concentration required for 50% growth inhibition (IC_50_) has also been well determined for different types of cells [16,22]. Nevertheless, no data is available on the amount of MA that is incorporated to the interior of the cells or the amount that remains in the culture medium. This is because no method to determine the concentration of MA in the cells and in the culture medium has been previously established.

The aim of this work was to develop an experimental method capable of determining the MA and OA concentration incorporated into the culture cell and that remaining in cell-culture medium. Reverse-phase high-performance liquid chromatography (HPLC) coupled to mass spectrometry (MS) enables the accurate detection of low concentrations of MA such as are present within cells. Until now, the methods used in different plant samples or other biological samples have been based on HPLC-UV/vis or gas chromatography (GC)-MS [1,23,24,25]. With these procedures the small amounts of MA present in cultured cells cannot be precisely determined.

The procedure described by us on cultured cells incubated with different concentrations of MA reveals the dynamics of MA incorporation into cells. This provides information on the cell-transport mechanism responsible for MA incorporation into the cells, and enables us to re-formulate the cell-survival curves for the different types of cell lines.

This study was conducted on two cell lines: HT29 an experimental model to study colon adenocarcinoma, and HepG2 an experimental model to study hepatic carcinoma. The tumorigenic capacity of HT29 cells has been shown in nude mice, in which they form moderately well-differentiated adenocarcinomas consistent with primary colon cancer (grade II). Doubling time is around 62 h. (ATCC: HTB-37). The line is positive for the expression of oncogenes: p53−, ras+, myc+, TGF b−, TGF a+ [16].

HepG2, a human hepatoma cell line, is considered a good system to study the *in vitro* metabolism of xenobiotics and liver toxicity [26]. They also constitute a good tool to investigate the cytoprotective, genotoxic, and antigenotoxic effects of compounds, to understand hepatocarcinogenesis, and to study drug targeting [27,28].

## 2. Results and Discussion

### 2.1. Growth Inhibitory Effects of Maslinic/Oleanolic Acid on HT29 and HepG2 Cancer Cells

**Figure 1:**
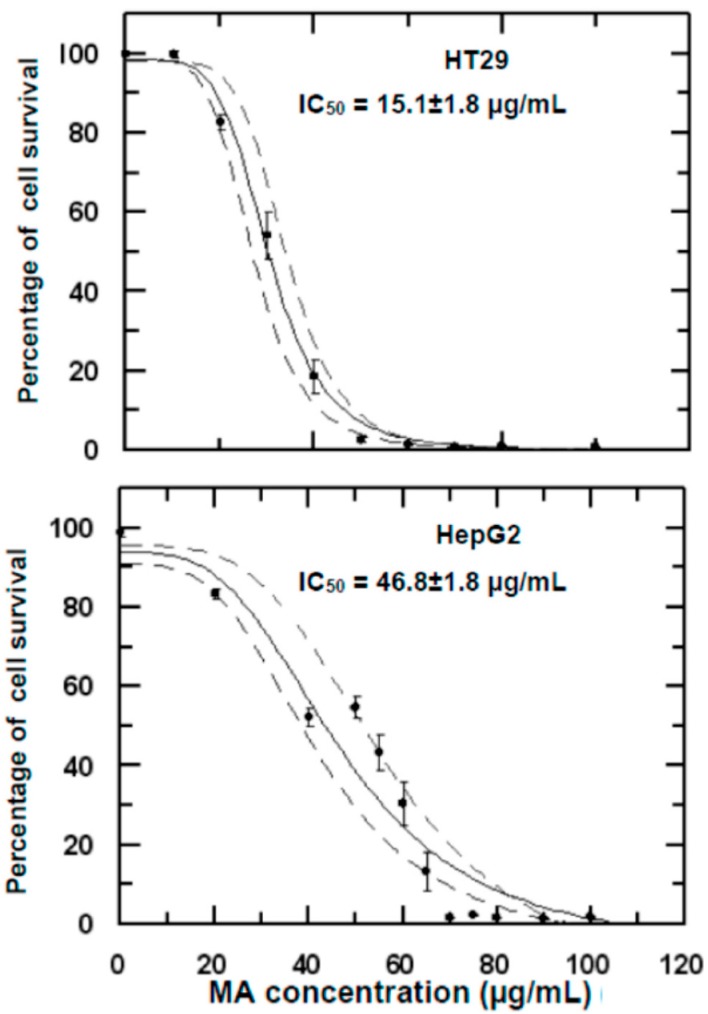
Growth-inhibitory effects of maslinic acid:oleanolic acid (98:2, *w*:*w*) on HT29 and HepG2 human cancer cells. Exponentially growing cells were treated with the concentration of MA. Cell-growth inhibition was analyzed by the MTT assay. The assays were performed using five replicates repeated five times. Results are mean ± SEM.

We examined the effect of the mixture of MA and OA (98:2, *w*:*w*) on the proliferation of HT29 and HepG2 cancer-cell lines using the MTT assay. HT29 and HepG2 cells were treated with increasing doses of this mixture. Their viability was determined by formazan dye uptake and expressed as a percentage of untreated control-cell proliferation (Figure 1). MA:OA (98:2, *w*:*w*) induced a dose-dependent decrease in viable formazan-accumulating cells after 72 h of treatment, ranging from 0 to 60 µg/mL and 0 to 120 µg/mL from HT29 and HepG2, respectively. The concentration required for 50% growth inhibition (IC_50_) was 15.1 ± 1.8 µg/mL (32.1 ± 0.1 µM) for HT29 and 46.8 ± 1.8 µg/mL (99.9 ± 0.1 µM) for HepG2.

These results agree with previous findings of our group [16] and Yap *et al*. [22] which demonstrate that MA inhibits proliferation of HT29, Caco-2, and Raji cells. Moreover, the results of the present work constitute the first evidence that MA:OA (98:2, *w*:*w*) also inhibits the cell proliferation of the HepG2 human cell line. This effect extends its anti-proliferative activity to tumor hepatic cell line with the peculiarity that the IC_50_ value is higher for HepG2 than in HT29. These results indicate that the sensitivity towards MA:OA varies for different types of cells. A 50% decrease in the viability of HepG2 cells required concentration more than three-fold higher than that needed to cause the same effect in HT29 cells.

### 2.2. Incorporation of MA and OA to the Inner of HT29 and HepG2 Cancer Cells

In the Experimental Section we describe an appropriate method for quantifying triterpenic acids (MA and OA) inside the cell and in the culture medium of HT29 and HepG2 cell cultures. We optimised an appropriate extraction method of these compounds and a HPLC-MS procedure that enables the quantification of small quantities of triterpenic acids in culture cells. Although HPLC-UV/vis [1,23,25], HPLC-MS/MS [29], or GC-MS [30] have been traditionally used for the analysis of plant or other samples, this is the first method described for the quantification of MA in cell-culture samples. Along the procedure, a little amount of MA and OA is lost due to the nature of a multi-stage procedure and the low quantities of these compounds presented in the samples. In this work we assume that MA and OA are recovered without been metabolized by the cell. No data about the transformation or metabolism of MA and OA by HT29 and HepG2 cells exist. In these types of samples the low concentration of triterpenic compounds required an analysis method with a high resolution power. The procedure described enabled the precise separation and quantification of the MA and OA concentrations in the two types of samples: Cells and culture medium. A lineal relationship was found between the concentration of both acids and the peak area. The concentration of the experimental samples was between the concentrations used for the calibration curves. The signal level in the samples was appropriate for the accurate quantification of both compounds in both types of samples. To have a good method for the extracting and quantifying MA inside cells is very important to know fundamental aspects of the incorporation and the destination of this compound in the cells.

**Figure 2:**
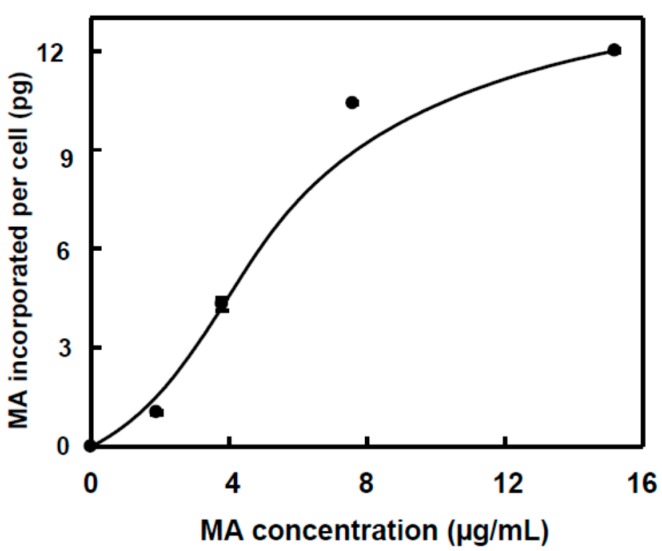
Effect of MA concentration on the incorporation of MA into HT29 culture cells. The amount of MA incorporated per cell was determined as the ratio between the amount of total MA detected in the cells and the number of cells estimated in culture (1,500,000 cells). A potential relationship was found between the MA concentration (*x*) and the amount incorporated into each cell (*y*), defined by the equation: *y* = 0.844 *x*^1.007^ (*r* = 0.96, *p* < 0.05).

Table 1 shows the amount of MA measured in the culture medium and inside the HT29 and HepG2 cells after incubation with different MA concentrations. For HT29, as the MA amount increased in the cell medium from IC_50/8_ to IC_50/2_, a significant increase was found in the MA incorporated into the cells. When the concentration was equivalent to IC_50_, the MA incorporated into the cells did not significantly differ from that found at the IC_50/2_ concentration. When the results were expressed as pg of MA incorporated per cell, a potential relationship was identified, similar to sigmoid kinetics (Figure 2), with an initial stage giving a slow response, a subsequent stage giving a higher response, and a final stage giving a saturation response. This indicates a possible cooperation mechanism in the incorporation of MA into the HT29 cells. With respect to the amount of MA added to the culture, the percentage of MA incorporated into the cells changed from 7% in the case of IC_50/8_ concentration to 17.2% in the case of the IC_50/2_ concentration. The MA-incorporation curve and the low percentage of MA incorporated into HT29 cells suggest that a specific receptor-carrier may exist in the membrane of the cells that at low concentrations joins the MA with low affinity, and, as the MA concentration increases, a cooperative behavior augments the entry of MA into the cell. The maximum level of transport is determined by the number of molecules of receptor-carrier present into the plasma membrane of these types of cells.

**Figure 3:**
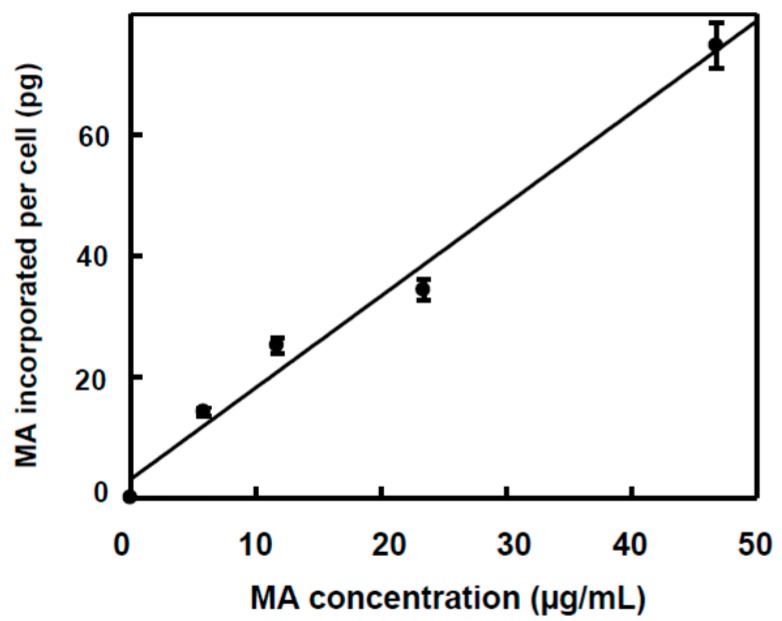
Effect of MA concentration on the incorporation of MA into HepG2 culture cells. The amount of MA incorporated per cell was determined as the ratio between the amount of total MA detected into the cells and the number of cells estimated in culture (2,000,000 cells). A linear relationship was found between the MA concentration (*x*) and the amount incorporated to each cell (*y*), defined by the equation: *y* = 1.516*x* + 3.11 (*r* = 0.99, *p* < 0.05).

In the case of HepG2, a linear relationship was found between the MA concentration in the culture medium and MA incorporated into the cell (Table 1, Figure 3). The percentage of MA incorporated into the cells changed from 22.6%, in the case of IC_50/2_ concentration, to 37.5%, in the case of IC_50/8_ concentration. This change would be related to a different type of transport of MA in HepG2 with respect to HT29. This is a system that lineally responds to the MA concentration present in the culture medium, which does not show saturation signs. These facts agree with a direct diffusion of MA towards lipid bilayer.

**Table 1:** Maslinic acid (MA) detected in the medium and inside HT29 and HepG2 cells incubated with different MA concentrations *.

	C^+^	C^-^	IC_50/8_	IC_50/4_	IC_50/2_	IC_50_
HT29						
µg MA added	0	0	22.65	45.3	90.6	181.2
µg MA in the medium	0	0	17.5 ± 0.7 ^a^	29.8 ± 0.9 ^b^	36.6 ± 1.8 ^c^	117.0 ± 3.5 ^d^
µg MA inside cells	0	0	1.6 ± 0.1 ^a^	6.5 ± 0.3 ^b^	15.6 ± 0.8 ^c^	18.0 ± 0.9 ^c^
HepG2						
µg MA added	0	0	75.9	151.8	303.6	607.2
µg MA in the medium	0	0	56.5 ± 2.3 ^a^	129.0 ± 5.2 ^b^	179.3 ± 7.2 ^c^	407.5 ± 20.4 ^d^
µg MA inside cells	0	0	28.5 ± 1.4 ^a^	50.4 ± 2.5 ^b^	68.7 ± 3.4 ^c^	149.6 ± 7.5 ^d^

* Six different experimental groups were made: C^+^, control with foetal bovine serum; C^-^, control without foetal bovine serum; IC_50/8_, IC_50/4_, IC_50/2_, IC_50_, refer to cell culture incubated with a MA concentration equivalent to IC_50/8_, IC_50/4_, IC_50/2_ and IC_50_, respectively. Results are means ± SEM of five independent data. Values followed by different letters are significant (*p* < 0.05) different.

HT29 cells have many characteristics of enterocytes and HepG2 cells have many characteristics of hepatocytes. The results of this work demonstrate a different cellular bioavailability and sensitivity to MA in both types of cells. Enterocytes have a higher sensitivity for MA and a lower transport capacity than do HepG2. These results reflect the need to study the dynamics of MA incorporation into the intestine and liver cells of a whole organism in order to learn more about the bioavailability dynamics of MA in both types of tissues.

Table 2 showed the amount of OA measured in the interior of HT29 and HepG2 cells after incubation with different concentrations of 98%-enriched MA solution. The OA detected in the interior of the cells comes from the OA present as contamination in the 98%-enriched MA solution. All the OA detected was present in the interior of the cells, while no OA was detected in the culture medium. As OA added to the culture increased, the amount of OA incorporated into the cell also increased. These results demonstrate that method is appropriate to determine the OA concentration in culture cells.

**Table 2:** Oleanolic acid (OA) detected in the medium and inside of HT29 and HepG2 cells incubated with different MA concentrations *.

	C^+^	C^-^	IC_50/8_	IC_50/4_	IC_50/2_	IC_50_
HT29						
µg OA added	0	0	0.47	0.93	1.85	3.70
µg OA in the medium	0	0	0	0	0	0
µg OA inside cells	0	0	0.01 ± 0.01 ^a^	0.50 ± 0.02 ^b^	1.00 ± 0.05 ^c^	2.17 ± 0.11 ^d^
HepG2						
µg OA added	0	0	1.52	3.04	6.07	12.14
µg OA in the medium	0	0	0	0	0	0
µg OA inside cells	0	0	1.16 ± 0.06 ^a^	4.57 ± 0.23 ^b^	7.19 ± 0.35 ^c^	7.88 ± 0.39 ^c^

* Six different experimental groups were made: C^+^, control with foetal bovine serum; C^−^, control without foetal bovine serum; IC_50/8_, IC_50/4_, IC_50/2_, IC_50_, refer to cell culture incubated with a MA concentration equivalent to IC_50/8_; IC_50/4_, IC_50/2_; and IC_50_, respectively. Results are means ± SEM of five independent data. Values followed by different letters are significant (*p* < 0.05) different.

In this work we described, for the first time, an appropriate HPLC-MS method for determining the MA and OA concentration in culture media and inside of cultured cells. The application of this method in HT29 and HepG2 reveals the MA and OA availability and the specific dynamics of entry into the cells.

## 3. Experimental Section

### 3.1. Materials and Chemicals

The main chemicals and materials used for cell culture were: Dimethylsulfoxide (DMSO), Dulbecco’s modified Eagle’s medium (DMEM), Minimum Essential Medium (MEM), foetal bovine serum (FCS), penicillin/streptomycin, phosphate buffered saline (PBS), 3-(4,5-dimethylthiazol-2yl)-2,5-diphenyltetrazolium bromide (MTT) all purchased from Sigma (St. Louis, MO, USA) culture flasks, and well plates (Techno Plastic Products, Trasadingen, Switzerland).

The MA-enriched extract was kindly donated by Drs. Andrés García-Granados and Andrés Parra, of the Department of Organic Chemistry, University of Granada, Spain. It was obtained from olive pomace, using the method described in patent number PCT/ES97/000190. It is a powder comprising 98% MA and 2% OA, which is stable when stored at 4 °C. MA was dissolved before use at 10 mg/mL in 50% DMSO and 50% PBS. DMSO concentration in contact with cells ranged from 0.009% to 0.075% for HT29 and from 0.029% to 0.234% for HepG2. Stock solution was frozen and stored at −20 °C. For treatments, this solution was diluted in cell-culture medium. OA used as standard was purchased from Sigma Chemical Co. (St. Louis, MO, USA). The rest of chemicals and solvents used were obtained of the best quality commercial sources.

### 3.2. Cell Culture

Human HT29 (ECACC no. 91072201) and human HepG2 (ECACC no. 85011430) cell lines were provided by the cell bank of the University of Granada, Spain. HT29 cells were cultured in DMEM, supplemented with 4.5 g/L glucose, 2 mM glutamine, with and without 10% heat-inactivated FCS, 10,000 units/mL penicillin, and 10 mg/mL of streptomycin. HepG2 cells were cultured in MEM medium, supplemented with Eagle’s salts and 2 mM glutamine, at 37 °C in an atmosphere of 5% CO_2_ and 95% of humidity. Subconfluent monolayers of cells were used in all experiments.

### 3.3. Determination of the Effect on HT29 and HepG2 Cell Viability by MTT Assay

To determine the effects on cell viability, we incubated HT29 and HepG2 cells with different MA concentrations for 72 h. The MA concentrations ranged from 0 to 60 µg/mL of medium and from 0 to 120 µg/mL of medium for HT29 and HepG2 cells, respectively.

Cell viability was determined by measuring the absorbance of MTT dye staining of living cells, as described Matito *et al*. (2003) [31]. For this assay, 1.5 × 10^4^ HT29 cells/well and 2.0 × 10^4^ HepG2 cells/well were cultured on 96-well plates.

Concentrations that inhibited cell growth by 50% (IC_50_) were calculated based on the survival rate compared with untreated cells. Relative cell viability was measured by the absorbance on an ELISA plate reader (Tecan Sunrise MR20-301, TECAN, Grödig, Austria) at 550 nm.

### 3.4. Extraction of Maslinic and Oleanolic Acid from HT29 and HepG2 Culture Cells and Medium

HT29 and HepG2 were incubated in a medium containing MA (98% purity) at their IC_50_, IC_50/2_, IC_50/4_, IC_50/8_ concentration values.

After treatment, the medium was removed from the plates and kept in Falcon tubes. Cells were washed three times with phosphate buffer solution (PBS) and collected with a scraper in 1 mL of PBS. This cell solution was placed in previously weighed Eppendorf tubes. The tubes were centrifuged at 1000× *g* for 5 min at 4 °C, the supernatant was removed, and the tube containing the cells was again weighed. After adding 0.5 mL of lysis buffer containing 20 mM Tris-HCl pH 7.5, 1 mM dithiothreitol (DTT), 1 mM ethylenediaminotetracetic acid (EDTA), 2% Triton X100, 0.2 mM phenyl methylsulfonyl fluoride (PMSF) and 2% sodium deoxycholate the cell suspension was sonicated for 5 min in water and ice.

Falcon tubes containing the medium and the Eppendorf tubes containing the cells were centrifuged at 1000× *g* for 5 min at 4 °C. One mL of ethyl acetate was added to 0.5 mL of the supernatant of each sample. This mixture was vigorously stirred by Vortex for 1 min and centrifuged at 6500× *g*, for 5 min at 20 °C. The upper organic fraction was collected in a 15 mL vial, and the remaining fraction was re-extracted five more times. The pool of all the upper organic fractions was desiccated in a rotary evaporator (130 mbar and 40 °C). The resulting residue was dissolved in 0.5 mL of methanol, filtered through a 0.2 µm syringe membrane and used for the HPLC-MS and HPLC-MS/MS analysis.

### 3.5. HPLC-MS and HPLC-MS/MS Analysis

HPLC-MS and HPLC-MS/MS analysis of MA and OA was performed using a procedure previously described 1, with some modifications. A HPLC Agilent Series 1100 (Agilent Technologies, Santa Clara, CA, USA) system consisting of vacuum degasser, autosampler, a quaternary pump, a diode-array detector, and an Esquire 6000 ion-trap mass spectrometer (Bruker Daltonics, Billerica, MA, USA) equipped with an electrospray ionization (ESI) source operating in negative ion mode was used. Separation was achieved by isocratic elution using a reverse-phase Spherisorb ODS-2 (Waters Corporation, Milford, CT, USA) (25 cm–4.6 mm, 5 μm) column. The injection volume was 2 μL. The solvent used for separation was methanol:water with 0.1% formic acid (pH = 3.1) at a proportion of 92:8 (*v*:*v*). A flow rate of 0.8 mL·min^−1^ and a temperature of 35 °C were used. Ions were detected in an ion-charged control (ICC) (target: 2500 ions) with an accumulation time of 170 ms, using the following operation parameters: capillary exit voltage (fragmenter): −300.0 V; capillary voltage: 4000 V; nebulizer pressure: 60 psig, drying gas: 11 L·min^−1^, gas temperature: 350 °C. This chromatographic system operates with Bruker Daltonics Data Analysis Software (Bruker Daltonics). The fragmentation options used for the MS/MS analyses were: Energy 0.8 V, width *m*/*z* = 10, and time 40 ms.

**Figure 4:**
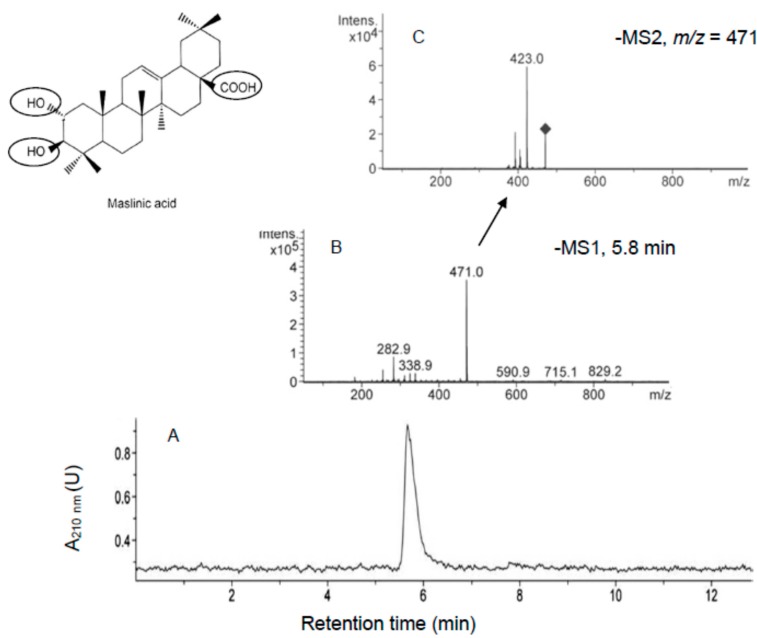
HPLC-UV/vis (**A**); HPLC-MS (**B**); and HPLC-MS/MS (**C**) of maslinic acid (MA, mol mass: 472). Two microlitres of sample was subjected to HPLC with a Spherisorb ODS-2 column that was eluted with methanol-water. Panel **A** shows the chromatogram for reading the absorbance at 210 nm; Panel **B** shows the MS1 spectrum of MA peak; Panel **C** shows the MS2 spectrum for *m*/*z* = 471.

Typical chromatograms for standard MA are shown in Figure 4. This compound is characterized by a retention time of 5.80 ± 0.01 min (Panel A). HPLC-MS analysis showed a peak of *m*/*z* = 471.0 corresponding with the negative ion of MA (Panel B). The HPLC-MS/MS analysis showed major ions of *m*/*z* = 423, 393, 405 (Panel C).

**Figure 5:**
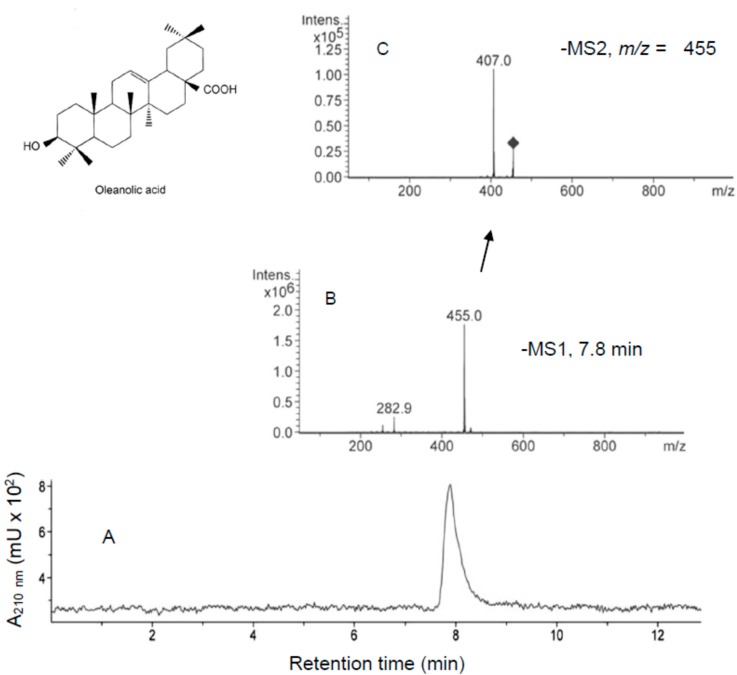
HPLC-UV (**A**); HPLC-MS (**B**); and HPLC-MS/MS (**C**) of oleanolic acid (OA, mol mass: 456). Two microlitres of sample was subjected to HPLC with a Spherisorb ODS-2 column that was eluted with methanol-water. Panel **A** shows the chromatogram for reading the absorbance at 210 nm; Panel **B** shows the MS1 spectrum of OA peak; Panel **C** shows the ion 455 MS2 spectrum.

Typical chromatograms for standard OA are shown in Figure 5. This compound is characterized by a retention time of 7.8 ± 0.01 min (Panel A). HPLC-MS analysis showed a peak of *m*/*z* = 455.0 corresponding with the negative ion of OA (Panel B). The HPLC-MS/MS analysis showed major ions of *m*/*z* = 407, 405, 391, 282.9, 254.8 (Panel C).

Due to their chromatographic behavior, MA and OA can be detected and quantified on the basis of retention time and the presence of 471 and 455 ion and their integrated areas, respectively. Standard calibration curves were constructed with 10 concentrations of MA (from 0.0005 to 0.01 mg·mL^−1^) and OA (from 0.01 to 0.05 mg·mL^−1^) standards. The equations formulated relating concentration (*x*, mg·mL^−1^) and peak areas (*y*, arbitrary units) were: *y* = 1,242,217,322*x* + 178,692 for MA; y = 537,528,522*x* + 2,449,795 for OA. In all cases *r*^2^ was higher than 0.98.

**Figure 6:**
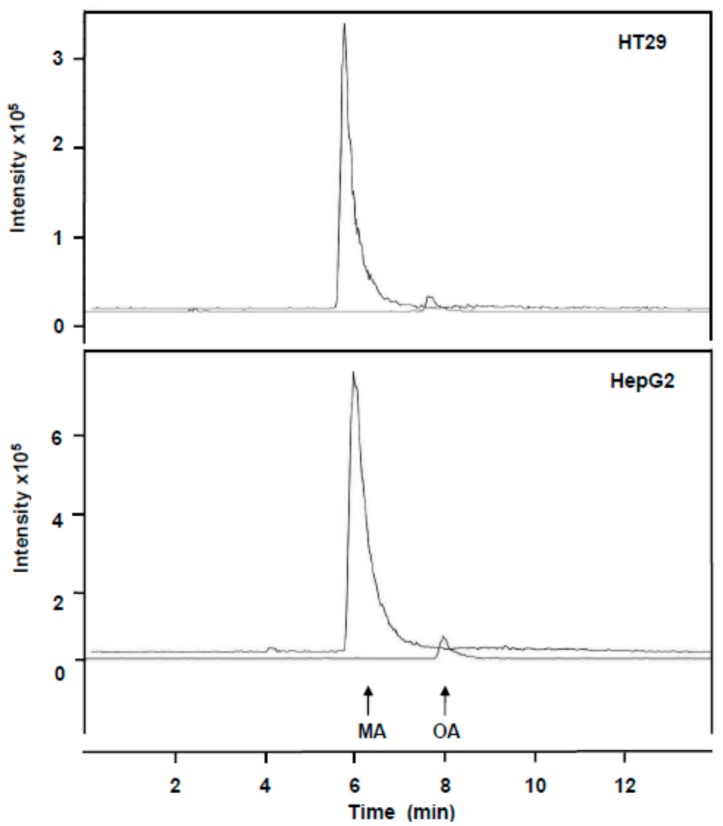
HPLC-MS analysis of HT29 and HepG2 cell extracts after incubation with maslinic acid (98% purity) at a concentration equivalent to IC_50_ and IC_50/4_, respectively. The chromatograms for *m*/*z* = 471 (MA) and 455 (OA) ions are shown on separate lines. The concentrations of MA and OA detected in HT29 were 8.38 and 0.92 µg/mL, respectively. The concentrations of MA and OA detected in HepG2 were 16.71 and 0.89 µg/mL, respectively.

We used HPLC-MS to determine the concentrations of MA and OA in cultured HT29 and HepG2 cell samples and medium. Thus, the presence and concentration of MA and OA in both types of samples was determined based on the peak area of *m*/*z* = 471 and 455 ion, respectively. Figure 6 shows the typical chromatograms of treated HT29 and HepG2 cell extracts. In this figure the chromatograms obtained for *m*/*z* = 471.1 and 455.1 ions are shown. The presence of the 455.1 ion is due to the presence of OA contained in the MA standard used.

### 3.6. Statistical Treatment

The results are expressed as mean ± SEM. Data were analysed by one-way analysis of variance. Differences between means were analyzed by an unpaired Student’s *t*-test. Linear correlations were determined by least-squares regression analysis. The criterion of significance was taken as *p* < 0.05.

## Abbreviations

DMEM, Dulbecco’s modified Eagle’s medium; FCS, foetal bovine serum; GC, gas chromatography; HPLC, high-performance liquid chromatography; IC_50_, concentration required for 50% growth inhibition; MS, mass analysis; MA, maslinic acid; MEM, Minimum Essential Medium; MTT, 3-(4,5-dimethylthiazol-2yl)-2,5-diphenyltetrazolium bromide; OA, oleanolic acid; PBS, phosphate buffered saline; SEM, standard error of the mean.
